# High human blood meal index of mosquitoes in Arba Minch town, southwest Ethiopia: an implication for urban mosquito-borne disease transmission

**DOI:** 10.1007/s00436-024-08121-4

**Published:** 2024-01-18

**Authors:** Adisu Akirso, Girum Tamiru, Nigatu Eligo, Bernt Lindtjørn, Fekadu Massebo

**Affiliations:** 1 Department of Biology, Arba Minch University, Arba Minch, Ethiopia; 2 Centre for International Health, University of Bergen, Bergen, Norway

**Keywords:** Bovine blood meal index, Circum-sporozoite protein, Human blood meal index, Urban mosquitoes

## Abstract

Unplanned human population shifts in urban areas are expected to increase the prevalence of vector-borne diseases. This study aimed to investigate mosquito species composition, blood meal sources, and malaria vectors in an urban area. Indoor-resting adult mosquitoes were collected using Prokopack and host-seeking mosquitoes using Centers for Disease Control and Prevention light traps in Arba Minch town. Larval collection from artificial containers was done in those houses selected for adult mosquito collection. *Anopheles* adults collected and emerged from larvae were identified morphologically using a taxonomic key. ELISA was used to identify blood meal sources in freshly fed *Anopheles* and *Culex* mosquitoes, and CSP of *Anopheles* mosquitoes. A total of 16,756 female mosquitoes were collected. Of these, 93% (15,571) were *Culex*, 6% (1016) were *Anopheles*, and 1% (169) were *Aedes* mosquitoes. Out of the 130 adult mosquitoes that were raised from larvae collected from the containers, 20% were *An. rhodesiensis*, while the remaining 80% were *Aedes* mosquitoes. Out of 823 mosquitoes tested for blood meal origins, 86.3% (710/823) tested positive for human blood, 2.2% (18/823) tested positive for bovine blood, and 11.5% (95/823) were negative for human and bovine antibodies. *Anopheles gambiae* complex had a human blood meal index (HBI) of 50% (90/180; CI 42.3–57.5%) and a bovine blood meal index (BBI) of only 0.5% (95% CI 0.01–3.1%). *Culex* HBI was 96.7% (620/641), and its BBI index was 2.4% (15/641). While it was low (0.8%) in *Culex*, the proportion of *An. gambiae* complex with unidentified blood meal sources was 49.5% (95 CI% 41.9–56.9%). Among the 1016 *Anopheles* mosquitoes tested, a single *An. gambiae* complex (0.1%; 1/1016) was positive for *P. vivax* CSP. The high HBI indicates frequent contact between humans and vectors. To reduce human exposure, personal protection tools should be implemented.

**Supplementary Information:**

The online version contains supplementary material available at 10.1007/s00436-024-08121-4.

## Introduction

Over half of the world’s population is at risk of vector-borne diseases (World Health Organization 2014). Urban malaria cases account for between 6 and 28% of annual malaria cases worldwide (Keiser et al. 2004). This number could increase because of population migration to urban settings, and it remains a concern in the context of malaria elimination efforts (De Silva and Marshall 2012).

Rapid population growth in urban areas may change the epidemiology of malaria in African cities (Hay et al. 2005; World Health Organization 2022). In the past, population movement has contributed to the spread of the disease and the campaign’s failure to eliminate malaria. Cities may suffer from “ruralisation,” which occurs when migrants bring their rural habits and practices to urban areas, such as livestock husbandry, traditional water usage and storage methods (wells), agriculture, and food production, which may offer breeding grounds for mosquitoes (Keiser et al. 2004; Hay et al. 2005; World Health Organization 2022). Irrigated urban agriculture, which has helped to alleviate poverty and increase food security in rapidly urbanizing sub-Saharan Africa, may inadvertently support malaria vectors (Klinkenberg et al. 2008). In addition, rural malaria control strategies cannot be transferred directly to urban areas (World Health Organization 2022). Designing appropriate interventions requires a better understanding of the vectors’ biology and behaviour in towns.

The three most common malaria vectors in Africa are *An. gambiae*, *An. arabiensis*, and *An. funestus* (Doumbe-Belisse et al. 2021). In Ethiopia, *An. arabiensis* is the primary vector for malaria in both rural and urban areas, while *An. pharoensis* is a secondary vector (Animut et al. 2013; Abraham et al. 2017; Esayas et al. 2020). In urban Ethiopia, notably in Eastern parts, the new species *An. stephensi* has been identified (Carter et al. 2018; Balkew et al. 2020). It is a potent vector for *P. vivax* and *P. falciparum* transmission in Asia and the primary malaria vector in India and the Persian Gulf (Sinka et al. 2011). This mosquito is highly adapted to typical urban conditions, and its presence can pose a threat to malaria control efforts, especially in urban areas (Balkew et al. 2020; Tadesse et al. 2021). Additionally, this species has developed resistance to various insecticides such as DDT, pirimiphos-methyl, propoxur, bendiocarb, and deltamethrin (Yared et al. 2020), which makes it challenging to prevent its spread to new locations.

Although several studies have described the entomological indicators of malaria-transmitting mosquitoes in rural areas (Massebo et al. 2013a; Abraham et al. 2017; Esayas et al. 2020), there is a need to investigate urban mosquito species, blood-feeding patterns, and malaria transmission vectors. The blood meal sources of mosquitoes can indicate their potential to transmit pathogens. Mosquitoes that frequently bite humans are more likely to transmit pathogens than those that infrequently visit human dwellings and bite humans (Sherrard-Smith et al. 2019). To effectively design and implement interventions, it is crucial to understand blood-feeding patterns, species composition, and primary vectors.

Urban areas may see increased malaria and other vector-borne diseases due to poor housing, sanitation, water management, healthcare, and economic disparities. Those who inhabit the outskirts of towns may be at the greatest risk due to the aforementioned factors. Arba Minch is a malaria-endemic town in Ethiopia, where both *Plasmodium falciparum* and *P. vivax* are common parasites and *An. arabiensis* is the principal vector (Getawen et al. 2018). Various mosquito breeding sites may contribute to the year-round transmission of malaria. A better understanding of species composition, blood-feeding behaviour, and breeding habitats may be necessary to adapt strategies against present and emerging urban vectors. Thus, this study provides information about the key entomological indicators, including species composition, feeding and resting densities, blood meal indexes, CSP infection rate, and entomological inoculation rates. The purpose of this study was to get a better understanding of urban malaria vector species, their feeding behaviour, and their role in malaria transmission.

## Materials and methods

### Description of the study area

**Fig. 1:**
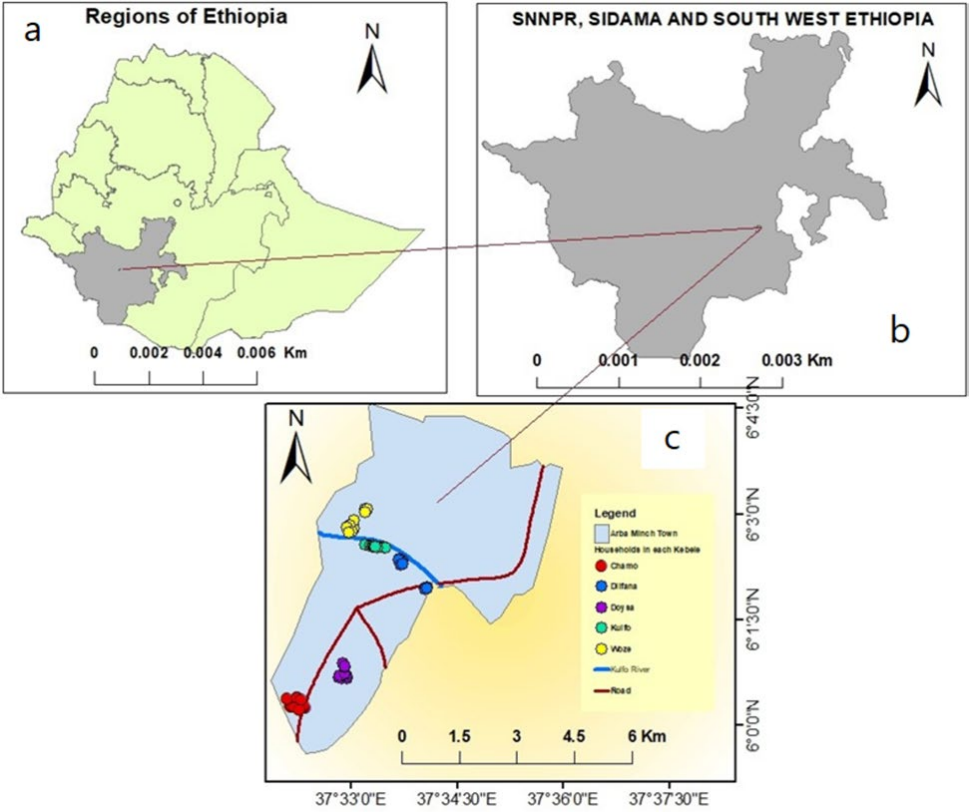
Map of the study area: **a** map of Ethiopia, **b** former Southern Nation and nationalities and people regional state, **c** Arba Minch town and study *Kebeles*

Arba Minch town is located in Ethiopia’s Southern Nations, Nationalities, and Peoples Regional State (SNNPRs) (Fig. 1). It is located 505 km to the southwest of the capital of Ethiopia, Addis Ababa. It comprises four administrative sub-cities: Secha, Sikella, Abaya, and Nechsar. The town has 16 *Kebeles* (the smallest administrative unit), which are all malarious. The average altitude of the study area was 1281 m above sea level. The average annual temperature is 29.7 °C, and the yearly rainfall is 900 mm.

The river Kulfo crosses the town on the northwest side and is being used for irrigation. There are many mosquito breeding sites at the edge of the river. The climate is favourable to the cultivation of different fruits and vegetables.

### Study design

**Fig. 2:**
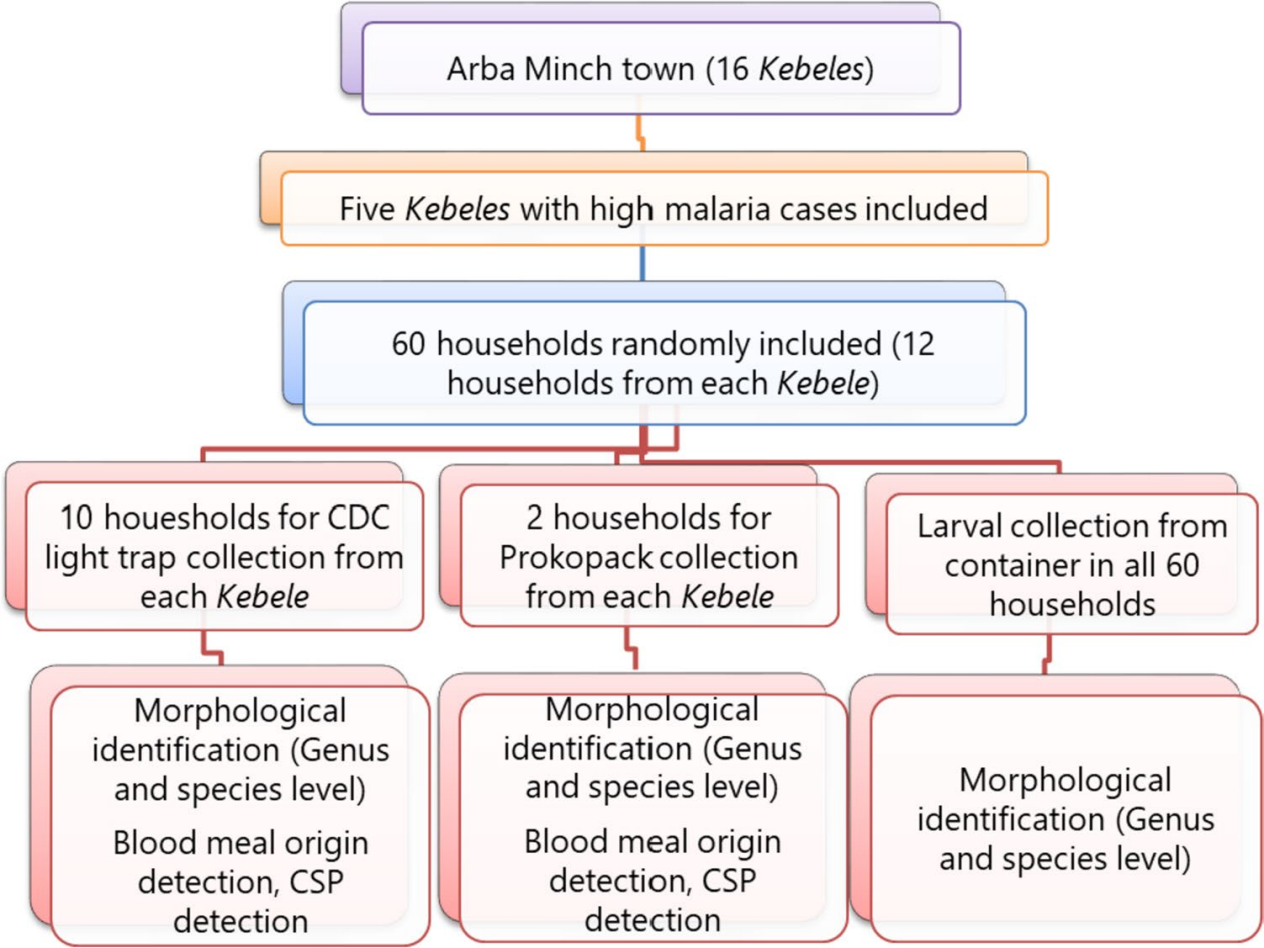
The study design flow chart

An entomological study was conducted in Arba Minch town from April to November 2022, except October. Five *Kebeles* (Dilfana, Kulfo, Woze, Chamo, and Doysa) were selected from 16 *Kebeles* based on the number of malaria cases. The three entomological sampling techniques employed were CDC light traps, Prokopacks, and container searching for larvae (Fig. 2).

Fifty houses were selected for the CDC light trap collection, with ten houses included from each of the five *Kebeles*. The first house was chosen randomly from one corridor, and the following houses were selected systematically. For the Prokopack aspirator collection, two houses were selected randomly from each *Kebele*. Additionally, *Anopheles* larvae and pupae were collected from artificial containers in the 60 houses selected for CDC light traps and Prokopack aspirator collection. All larval and pupal samples were reared in the laboratory at Arba Minch University until they emerged into adult stages and then identified using a morphological key (Coetzee 2020).

### Mosquito collection and processing

#### Larval collection

All artificial containers, including waste tyres, plastic storage for domestic and construction usage, and water storage, were inspected, and those that tested positive for mosquito larvae were sampled by dipping. Pupae and the last two instar larvae were collected and raised into adults in the Medical Entomology and Vector Control laboratory at 25 to 27 °C and 70–80% relative humidity. No additional food was provided to the larvae as they were collected from nutrient-rich habitats. Pupae were placed in cages for adult development and adults were provided with 10% sugar solution.

#### CDC light trap collection

Adult female *Anopheles* mosquito sampling was conducted using CDC light traps (Model 512; John W. Hock Company, Gainesville, FL, USA) once per month in 50 randomly selected houses. The traps were installed near the foot of the bed where the individual slept, about 1.5 m above the floor. Light traps were switched on at 6:00 pm and switched off at 6:00 am (WHO 1975).

#### Prokopack aspirator collection

Prokopack aspirator collection was done once a month in two homes in each *Kebele*. All selected houses conducted aspirations indoors, beginning at 6:00 am and ending at about 8:00 am. Mosquitoes were systematically aspirated on the walls and ceilings for 20–30 min per house once per month (Vazquez-Prokopec et al. 2009).

#### Morphological identification of mosquitoes

Alive mosquito killing was done by freezing. Anopheline and culicine were first identified. Speciation of *Anopheles* was done using a morphological key (Coetzee 2020). The abdominal classification was done under the dissecting microscope into four categories: unfed, freshly fed, half-gravid, and gravid. Female *Anopheles* mosquitoes were preserved individually in vials with silica gel for blood meal source assay and circum-sporozoite protein (CSPs) test. Each month, ten *Culex* mosquitos were collected from each *Kebele* and stored for blood meal analysis to obtain a representative sample.

#### Blood meal sources and CSP detection assays

The enzyme-linked immunosorbent assay (ELISA) was used to detect for human and bovine blood antigens following the procedure of Beier et al. (1988) and *P. falciparum* and *P. vivax*_210 CSPs following the protocol of Beier et al. (1987). The head and thorax of female *Anopheles* mosquitoes were used to test for *P. falciparum* and *P. vivax*_210 CSPs, while the abdomen of freshly fed *Anopheles* and *Culex* mosquitoes was tested for blood meals using anti-human and anti-bovine antibodies. The detailed description was indicated in previous publications (Massebo et al. 2013b, a).

### Statistical analysis

IBM SPSS 20 statistical software (SPSS International, Chicago, USA) was used to enter and analyse all data. The human blood index (HBI) and bovine blood index (BBI) were calculated as follows.

$$\mathrm{Human \;blood \;meal \;index}=\frac{n}{N} : n=\mathrm{number \;of \;human \;fed};\;N=\mathrm{number \;tested}$$

$$\mathrm{Bovine \;blood \;meal \;index}=\frac{n}{N}:n=\mathrm{number \;of \;cattle \;fed};\;N=\mathrm{number \;tested}$$

The percentage of mosquitoes that tested positive for *P. falciparum* and *P. vivax* out of all mosquitoes was used to determine the sporozoite rate. The *Plasmodium* EIRs were computed from CDC trap collections of mosquitoes using the following formula (Lines et al.^1991^):

$${\text{EIR}}=1.605\times \left(\frac{{\text{n}}}{{\text{N}}}\times \frac{{\text{ni}}}{{\text{Ni}}}\right)\times \mathrm{number \;of \;days \;a \;month},$$

where *n* is the number of CSP positive; *N* is the number of mosquitoes tested; ni is the number of mosquitoes collected; and Ni is the number of CDC light trap catches. An analysis of variance (ANOVA) was used to compare the monthly *Anopheles* mosquito population density. The months with the highest mean mosquito density were identified using Tukey’s honestly significant difference (HSD) test. Every test was run using a 0.05 threshold of significance.

## Results

### Mosquito species composition

Sixteen thousand seven hundred fifty-six adult female mosquitoes were collected using CDC light traps and Prokopack collection techniques. During the study period, the *Culex* mosquito predominated, accounting for 93% of the collected mosquitoes (15,571/16,756). The remaining mosquitoes were of the genera *Aedes* (1%, 169 mosquitoes) and *Anopheles* (6%, 1016 mosquitoes). Most of the mosquito larvae that developed into adults (80%, 104 out of 130) were of the *Aedes* species, while the remaining 20% (26 out of 130) were *An. rhodesiensis*, which is the only *Anopheles* species that was identified in the containers. Out of the 1016 adult *Anopheles* mosquitoes collected, 97% (986 out of 1016) were of the *An. gambiae* complex, 2.6% (27 out of 1016) were *An. rhodesiensis*, and 0.3% (3 out of 1016) were *An. pharoensis*.

### Monthly distribution of mosquitoes

Table 1 shows the seasonal abundance of mosquito species over 7 months and varied between months (*F* = 136.1, DF = 6, *P* < 0.001). *Anopheles* mosquitoes were found in higher numbers in September, with 2.4 *Anopheles* per Prokopack per night and 13.5 *Anopheles* per CDC light trap per night.

**Table 1:** Monthly distribution of *Anopheles* and *Culex* mosquitoes collected indoors from Arba Minch town, southwest Ethiopia: April to November 2022, except October

Month	CDC collection			Prokopack collection			Total (%)
	*Anopheles*	*Aedes*	*Culex*	*Anopheles*	*Aedes*	*Culex*	
Apr	5	2	1516	2	2	538	2065
May	2	9	1629	1	2	397	2040
June	13	14	1956	1	1	569	2554
July	20	2	2459	1	0	407	2889
Aug	58	7	1706	9	0	833	2613
Sep	679	98	1494	24	0	365	2660
Nov	195	32	1305	6	0	397	1935
Total	972	164	12,065	44	5	3506	16,756

### Distribution of mosquitoes in *Kebeles*

Sixteen thousand seven hundred fifty-six female mosquitoes were collected in five *Kebeles* of Arba Minch Town during the study period. The majority of them (35.6%; 5711 out of 16,756) were collected from Doysa *Kebele*, while the least (10.6%; 1769 out of 16,756) were found in Woze (Table 2). The difference in mosquito population density among the *Kebeles* was statistically significant (DF = 4, *F* = 4.1, and *P* = 0.003). The highest density of *Anopheles* mosquitoes was also found in Doysa Kebele (23.5%; 239 out of 1016).

**Table 2:** Distribution of *Anopheles* and *Culex* mosquitoes collected indoors from Arba Minch town, southwest Ethiopia

Kebeles	Mosquitoes collected			
	*Anopheles***,** *n* (*%)*	*Culex***,** *n* (%)	*Aedes***,** *n* (%)	Overall
Dilfana	219 (21.6)	1537 (9.9)	39 (23.1)	1795 (10.7)
Kulfo	166 (16.3)	2893 (18.6)	45 (26.6)	3104 (18.5)
Woze	168 (16.5)	1571 (10.1)	30 (17.8)	1769 (10.6)
Chamo	224 (22.0)	3859 (24.8)	41 (24.3)	4124 (24.6)
Doysa	239 (23.5)	5711 (36.7)	14 (8.3)	5964 (35.6)
Overall	1016 (6.1)	15,571 (92.9)	169 (1.0)	16,756 (100)

### Blood meal sources of mosquitoes

The overall human blood meal index of mosquitoes was 86.3% (710/823, 95% CI 83.7–88.5), while the bovine blood meal index was 2.1% (17/823, 95% CI 1.2–3.23). There were many unidentified blood meal origins (11.4%; 94/823, 95% CI 9.3–13.8). The mixed blood origin of human and bovine was low and accounted for only 0.1% (Table 3).

**Table 3:** Blood meal origins of *Anopheles* and *Culex* mosquitoes collected indoors from Arba Minch town, southwest Ethiopia

Mosquitoes tested	Number tested	Blood meal sources			
		Human, *n* (%)	Bovine, *n* (%)	Mixed, *n* (%)	Unidentified, *n* (%)
*An. gambiae s.l*	180	90 (50)	1 (0.5)	-	89 (49.5)
*An. pharoensis*	2	1 (50)	1 (50)	-	-
*Culex* species	641	620 (96.7)	15 (2.4)	1 (0.17)	5 (0.8)
Total	823	711 (86.4)	17 (2.1)	1 (0.1)	94 (11.4)

The human blood meal index of *An. gambiae* complex was 50% (90/180, 95% CI 42.3–57.5), but the bovine blood meal index was only 0.5% (95% CI 0.01–3.1). In *Culex* mosquitoes, the human blood index was 96.7% (620/641), while the bovine blood meal index was only 2.4% (15/641). The study also found that the proportion of *An. gambiae* complex with unidentified blood meal sources was 49.5% (95% CI 41.9–56.9), which was quite high. The *Culex* mosquitoes had a low percentage (0.8%) of unidentified blood meal sources (Table 3).

### Blood meal indices of mosquitoes in different *Kebeles*

**Fig. 3:**
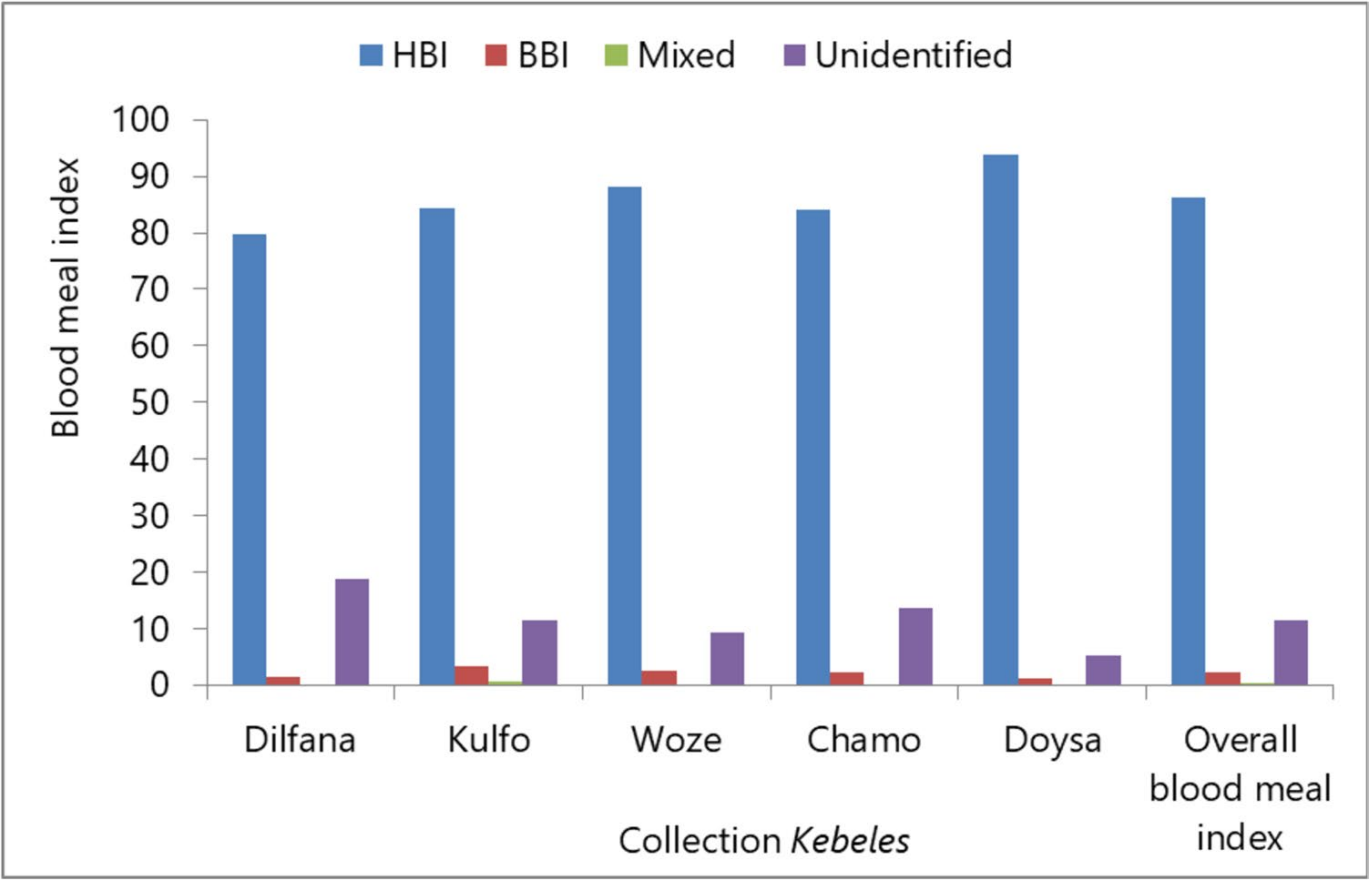
Overall blood meal index of mosquitoes collected by CDC light traps and Prokopack in Arba Minch town, southwest Ethiopia. HBI, human blood meal index; BBI, bovine blood meal index

The human blood meal index of mosquitoes ranged from the minimum of 79.7% in Dilfana to 93.7% in Doysa. The bovine blood meal index varied from 1.1% in Doysa to 3.4% in Kulfo. Mixed blood meal sources (human and bovine) were extremely rare, ranging from 0 in most *Kebeles* to 0.7% in Kulfo. In addition, the percentage of mosquitoes with an unidentified blood meal origin varied from 5.1% in Doysa to 18.8% in Dilfana (Fig. 3).

### Blood meal index of *Anopheles* species

**Fig. 4:**
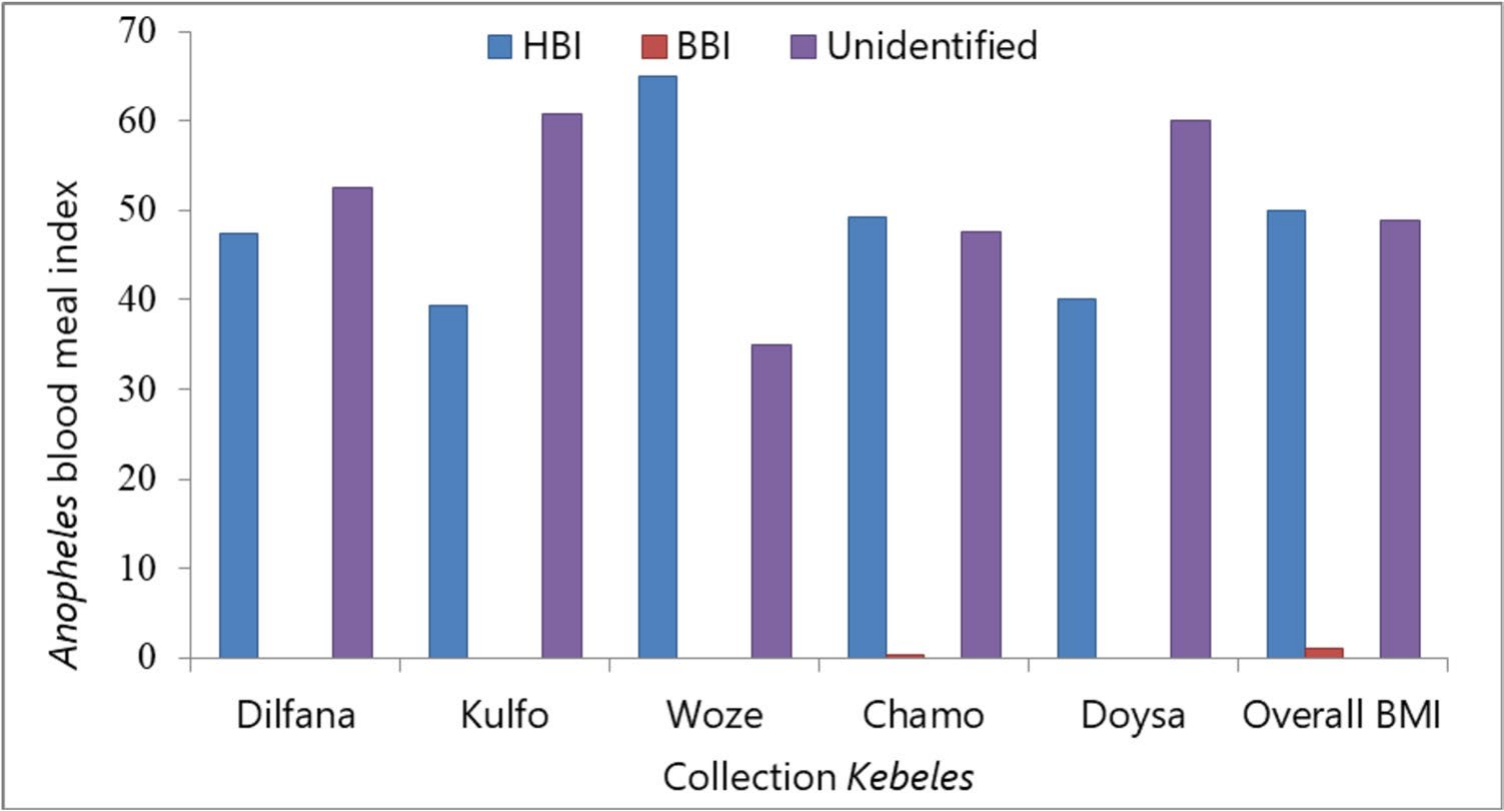
Blood meal index of *Anopheles* mosquitoes collected by CDC light traps and Prokopack in Arba Minch town, southwest Ethiopia. HBI, human blood meal index; BBI, bovine blood meal index

*Anopheles* species obtained blood meals from humans with indexes ranging from 39.3% in Kulfo to 65% in Woze. On the other hand, bovine blood meal indexes varied from 0% in most *Kebeles* to 0.3% in Chamo. The *Anopheles* species did not have any mixed blood origin. However, the unidentified blood meal index ranged from 35% in Woze to 60.7% in Kulfo (Fig. 4).

**Fig. 5:**
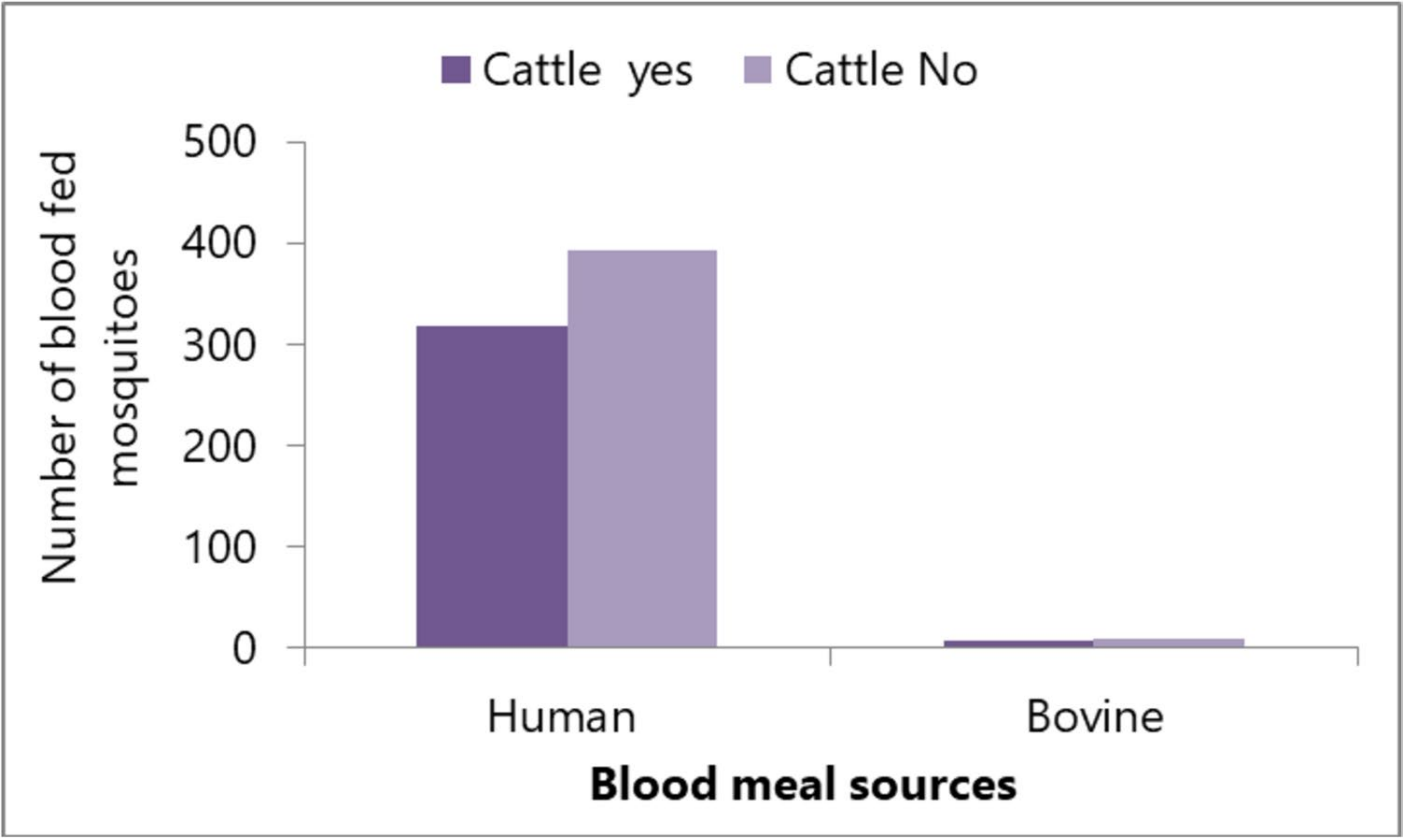
The number of human and bovine blood-fed mosquitoes collected using CDC light traps and Prokopack in households with and without cattle in Arba Minch town, southwest Ethiopia

Although the presence or absence of cattle in houses did not influence the blood meal index of mosquitoes, more mosquitoes with human blood meal origins were collected from houses without cattle (Fig. 5).

### CSP rate and EIR

A total of 1016 *Anopheles* mosquitoes were tested for *Plasmodium* circum-sporozoite proteins (CSPs). Of these, 97% (986/1016) were *An. gambiae* complex, 2.7% (27/1016) were *An. rhodesiensis*, and 0.3% (3/1016) were *An. pharoensis*. The only *P. vivax* sporozoite was detected in the *An. gambiae* complex. Overall, the *P. vivax* CSP rate was 0.1% (95% CI 0.003–0.5%), and the sporozoite rate of *An. gambiae s.l.* was 0.1% (95% CI 0.003–0.6). The monthly *P. vivax* sporozoite rate among the *An. gambiae s.l*. caught by the CDC trap was 0.15% (1/679) in September 2022 and zero for other months. The entomological inoculation rate (EIR) estimates varied from 0 (in most months) to 0.13% in September 2022.

## Discussion

Our research findings indicate that the majority of *Anopheles* and *Culex* mosquitoes fed on human blood, which suggests a higher risk of mosquito bites for humans. Several species of mosquitoes, including *An. gambiae* complex, *An. pharoensis*, *An. rhodesiensis*, *Aedes*, and *Culex*, were found in the study area. The *Anopheles gambiae* complex was identified as the primary vector for malaria in urban areas.

*Anopheles gambiae* complex was the primary mosquito responsible for carrying malaria in Arba Minch town. Both *P. falciparum* and *P. vivax* parasites have been detected in the *An. gambiae* complex in Arba Minch Getawen et al. 2018) and neighbouring areas (Massebo et al. 2013a; Abraham et al. 2017; Esayas et al. 2020). Unplanned urbanization, characterized by the development of slums, construction activities, and a lack of drainage systems, has led to the creation of numerous breeding habitats for *An. gambiae* complex in the urban environment (De Silva and Marshall 2012; Doumbe-Belisse et al. 2021). Besides the water bodies formed at construction sites in the town, the Kulfo River that passes through the town and flows into Lake Chamo may also provide breeding habitats for the mosquitoes. Thus, much work remains to be done in urban settings to mitigate malaria-related problems. Despite being a secondary malaria vector in Ethiopia, *An. pharoensis* is rarely sampled at the current study site (Abraham et al. 2017). This species was collected near the Kulfo River, where permanent water sources exist. *Anopheles rhodesiensis* could breed in containers like discarded plastics and barrels. At one of the study sites in Bahir Dar City, *An. rhodesiensis* was the most frequently captured species by CDC light traps, accounting for 90% of all collections (Getaneh et al. 2021). In contrast to the Bahir Dar study, where adult mosquitoes were caught using CDC light traps, we collected the larval stage of the species from containers at our study location.

C*ulex* mosquitoes were the most abundant throughout the study *Kebeles* and periods. The mosquito density was similar during the dry and rainy seasons and thus less likely to be related to rainfall. In most cities in Cameroon, *Culex* mosquitoes were the main species causing the highest nuisance to the population (Nchoutpouen et al. 2019). The dominant presence of *Culex* mosquitoes in this study area may be due to poor management of stagnant water sources. The rise in car washing stations, construction sites, and poor drainage systems might have contributed to this issue. To address this, a comprehensive strategy is needed.

The human blood meal index of *An. gambiae* complex was higher than the bovine meal index, indicating its higher opportunity to feed humans than animals. However, studies conducted in rural settings have reported different results. For instance, a study conducted in south-central Ethiopia reported similar proportions of human-fed and bovine-fed *An. arabiensis* (Animut et al. 2013). Another study conducted in southwest Ethiopia showed that *An. arabiensis* preferred bovine blood meal over human blood meal (Massebo et al. 2015). Although *Culex* mosquitoes feed on various blood sources worldwide (Zinser et al. 2004; Greenberg et al. 2013), in the study site, they have greater access to human blood than bovine. These mosquitoes often enter houses and feed on human blood, increasing the risk of transmitting diseases such as West Nile virus, lymphatic filariasis, and Rift Valley fever in urban areas (Zinser et al. 2004). Despite being a vector for several diseases, *Culex* mosquitoes have not received as much attention as *Anopheles* species in disease transmission. It is believed that this species contributes to the rapid growth and spread of many viral diseases (Riccetti et al. 2022).

In the current study, a significant number of *An. gambiae* complex mosquitoes had unidentified blood sources, indicating that they may have fed on different animals (Kent and Norris 2005). In southwest Ethiopia, 22.5% of *An. arabiensis* mosquitoes had unidentified blood sources (Massebo et al. 2013b), while in northern Ethiopia, the *An. gambiae* complex had unidentified blood sources comprising 42.8% of their total blood meals (Kindu et al. 2018). The limited number of antibodies used in the blood meal sources testing could be a contributing factor to this issue. Our study aimed to determine whether mosquitoes feed on humans or cattle by analysing their blood meals. We considered the results of a previous study conducted in nearby rural villages, which indicated that mosquitoes have a preference for feeding on cattle (Massebo et al. 2015). Additionally, animal composition in urban areas may differ from rural villages, contributing to unidentified blood meal sources. To address this issue, it is important to use a variety of antibodies for ELISA testing and PCR, along with diverse primers, to detect all blood meal sources of *Anopheles* mosquitoes accurately.

Out of all the *Anopheles* species that were tested for CSP, only one *An. gambiae* (probably *An. arabiensis*) complex tested positive for *P. vivax* CSP. However, the CSP rate was found to be lower than in a previous study conducted in the same town, where *P. falciparum* was the dominant parasite (Getawen et al. 2018). Another study conducted in a nearby rural village reported that both *An. gambiae* complex and *An. pharoensis* tested positive for CSP (Abraham et al. 2017). Despite the low CSP rate, *An. gambiae* complex, likely *An. arabiensis*, adapted to urban settings and persistently plays a role in malaria transmission. Although the EIR was calculated for *P. vivax*, which may not indicate active transmission, it is clear that *An. gambiae* complex plays a role in malaria transmission in urban areas. In a previous study conducted in the same town near Kulfo River, where malaria transmission is more intense compared to other areas, *An. arabiensis* was found to be carrying both *P. falciparum* and *P. vivax* parasites. The study also reported an P. falciparum EIR of up to 6.45 (Getawen et al. 2018). This implies that *An. arabiensis* is a well-adapted malaria vector in urban settings.

*Anopheles gambiae* complex was not molecularly identified. However, a previous study identified it as *An. arabiensis* (Massebo et al. 2013a), which is predominantly responsible for malaria transmission (Getawen et al. 2018). Moreover, the study was unable to identify other animal sources of blood meals for malaria vectors other than humans and cattle. Lastly, the study did not screen *Aedes* and *Culex* mosquitoes for filarial and arboviral infections.

## Conclusions

The study findings indicate that the *An. gambiae* complex is an efficient malaria vector dominant in Arba Minch town. These mosquitoes have a high tendency to feed on human blood, indicating frequent contact between humans and mosquitoes. Personal protection tools like bed nets and screening of houses could be implemented to minimize the human-vector contacts. The *Anopheles* mosquito identified as a container breeder was *An. rhodesiensis*, while *An. stephensi* was not found.

### Supplementary Information

Below is the link to the electronic supplementary material.

Supplementary file1 (XLSX 525 KB)

## Data Availability

The data used to draw a conclusion was presented in the article.
